# The impact of tertiary lymphoid structures on tumor prognosis and the immune microenvironment in non-small cell lung cancer

**DOI:** 10.1038/s41598-024-64980-y

**Published:** 2024-07-15

**Authors:** Yiming Weng, Jingping Yuan, Xue Cui, Jinsong Wang, Honglei Chen, Li Xu, Xinyi Chen, Min Peng, Qibin Song

**Affiliations:** 1 Department of Oncology, Renmin Hospital of Wuhan University, Wuhan, China; 2 Department of Pathology, Renmin Hospital of Wuhan University, Wuhan, China; 3 Department of Pathology, School of Basic Medical Sciences, Wuhan University, Wuhan, China

**Keywords:** Non-small cell lung cancer (NSCLC), Tertiary lymphoid structures (TLSs), Immunotherapy, Prognosis, Tumor microenvironment (TME), Cancer, Immunology

## Abstract

Non-small cell lung cancer (NSCLC) is a common malignancy whose prognosis and treatment outcome are influenced by many factors. Some studies have found that tertiary lymphoid structures (TLSs) in cancer may contribute to prognosis and the prediction of immunotherapy efficacy However, the combined role of TLSs in NSCLC remains unclear. We accessed The Cancer Genome Atlas (TCGA) and Gene Expression Omnibus (GEO) databases to obtain mRNA sequencing data and clinical information as the TCGA cohort, and used our own sample of 53 advanced NSCLC as a study cohort. The samples were divided into TLS+ and TLS- groups by pathological tissue sections. Patients of the TLS+ group had a better OS (*p* = 0.022), PFS (*p* = 0.042), and DSS (*p* = 0.004) in the TCGA cohort, and the results were confirmed by the study cohort (PFS, *p* = 0.012). Furthermore, our result showed that the count and size of TLSs are closely associated with the efficacy of immunotherapy. In addition, the TLS+ group was associated with better immune status and lower tumor mutation load. In the tumor microenvironment (TME), the expression levels of CD4+ T cells and CD8+ T cells of different phenotypes were associated with TLSs. Overall, TLSs are a strong predictor of survival and immunotherapeutic efficacy in advanced NSCLC, and T cell-rich TLSs suggest a more ordered and active immune response site, which aids in the decision-making and application of immunotherapy in the clinic.

## Introduction

Lung cancer is the leading cause of new cancer cases worldwide and the leading cause of cancer-related deaths, with 80% of lung cancer patients diagnosed with non-small cell lung cancer (NSCLC)^1^. As a common malignancy, the overall cure and survival rates of NSCLC have been very low, especially for patients in advanced stages, so its treatment and prognosis have been one of the hot topics of clinical research^2^. The advent of immunotherapy has brought new hope to the treatment of tumors and is one of the major advances in the clinical management of NSCLC recently. The first cancer type in solid tumors to be approved for immunotherapy, immune checkpoint inhibitors (ICIs) have greatly extended the survival of patients with advanced NSCLC and are their first-line standard of care option. Anti-PD-1/PD-L1 antibodies have become the widest-used anti-tumor immunotherapy, fighting tumors by revitalizing the function of T cells. However, due to the heterogeneity of tumors, only a small proportion of people show a durable response to ICI^3,4^. Therefore, further research is needed to investigate the mechanisms and applications of immunotherapy to provide more effective biomarkers for the treatment and prognostic assessment of NSCLCs.

Tertiary lymphoid structures (TLSs) are ectopic lymphoid tissues found around sites of chronic inflammatory diseases (such as tumors, infectious diseases, autoimmune diseases, and organ transplants) and play an important role in the adaptive immune response^5^. Aggregation of immune cells, mainly CD20+ B cell clusters, including T cell clusters, DC-Lamp+ dendritic cells (DCs), macrophages, and high endothelial venules (HEVs), to form tumor-associated TLSs^6–8^. In the tumor microenvironment (TME), TLSs provide a critical local microenvironment for immune cells and immune responses outside the lymphoid tissue and have been shown to be closely associated with tumor prognosis. The presence of TLSs was found to be an independent and good predictor in patients with grade 1 or 2 non-functional pancreatic neuroendocrine tumors (NFPanNETs)^9^. The presence of TLSs can also be an independent positive prognostic marker in patients with Oral squamous cell carcinoma (OSCC) containing T1–T4 stages^10^. TLSs are a strong prognostic factor in breast cancer, whether in untreated invasive primary tumors, in HER2+ breast cancer cohorts, or even in breast cancer cohorts that do not distinguish between the extent and stage of metastasis^11^. In a cohort of lung squamous cell carcinoma (LUSC), the germinal center (GC) was found to determine the prognostic value of TLSs^12^. The density of TLSs in synergy with tumor-infiltrating lymphocytes (TIL) in patients with stage II colorectal cancer predicts a better prognosis^13^. In a sarcoma cohort, B-cell enriched TLSs predict good prognosis and immunotherapeutic outcome^8^.

Thus, TLSs may be a predictor of cancer immunotherapy efficacy, but the lack of validated research evidence in NSCLC remains. Meanwhile, the mechanisms of interaction between TLSs and tumors and the crosstalk between immune cells in TLSs are unclear. Therefore, we divided the pathological section datasets from the CDSA archive in this study into TLS+ and TLS- groups by integrating them from the Cancer Genome Atlas (TCGA) database and according to the presence or absence of TLSs. We performed bioinformatic analyses of the TCGA cohort, including survival prognosis, gene mutation, copy number variation, immunogenicity, and TME cell infiltration landscape. Then, we collected pathological data from 53 patients with advanced NSCLC treated with ICIs at the Oncology Centre of the People’s Hospital of Wuhan University. TLSs and immune cells were analyzed by multiplex immunohistochemistry (mIHC) on a multi-spectral imaging system (Vectra 3.0) and analysis software (InForm 2.6) to elucidate the impact of TLSs expression in NSCLC on tumor prognosis and immunotherapy and to further explore the relationship between TLSs and immune cells in the TME.

## Materials and methods

### Ethics and patients

Patients diagnosed with advanced NSCLC were identified and the pathological diagnosis was confirmed by pathologists from the Wuhan University People’s Hospital. This retrospective study was approved by the Research Ethics Committee of the hospital. HE pathology stained sections of 53 patients prior to immunotherapy were obtained by pathology scanning software. Formalin-fixed paraffin-embedded (FFPE) blocks of 18 patients were retrieved and 4 µm-thick slides were taken for multiplex immunohistochemistry (mIHC).

Clinical data and pathologic specimens of NSCLC patients admitted to Wuhan University People’s Hospital from January 2018 to December 2021 were retrospectively collected. Inclusion criteria: (1) clear diagnosis of NSCLC by pathohistology; (2) TNM stage IIIb ~ IV; (3) the treatment regimen must include an immune checkpoint inhibitor; and (4) all patients received at least 2 cycles of drug therapy (21 days as a cycle). Exclusion criteria: (1) no clear pathological diagnosis; (2) NSCLC patients with TNM stage I-IIIa; (3) advanced NSCLC not treated with PD-1 inhibitors (4) less than 2 cycles of the target drug.

### Acquisition of non-small-cell-lung cancer datasets

The TCGA database (https://portal.gdc.cancer.gov/) was used to get the HE pathologically stained sections, mutation, and clinical data. There are 914 samples in the Cancer Digital Slide Archive (CDSA).

### Evaluation of TLSs in pathological sections

We evaluated the density of lymphocyte infiltration by HE pathologically stained sections of the TCGA cohort and study cohort. This study used methods based on this definition^14^ for counting all forms of TLS as follows: (1) lymphocyte aggregates (Agg) with lymphocyte infiltration but no lymphoid follicle formation; (2) primary follicles (FL-I), with well-defined clusters of round or oval lymphocytes or plasma cells (no germinal centers present); and (3) secondary follicles (FL-II), with well-defined clusters of round or oval lymphocytes or plasma cells (germinal centers present).

In our study, TLSs+ is defined as at least 1 lymphocyte aggregation structure found in the HE section of the subject, and the opposite is defined as TLSs-.

### Analysis of gene expression differences

To explore variances in gene expression between the two cohorts, we selected tumor tissue samples with both hematoxylin and eosin (H&E) staining slides and RNAseq expression data. These samples were then grouped based on the presence or absence of tertiary lymphoid structures (TLS), designated as TLS+ or TLS-, respectively. Differential expression analysis was performed using the R package “edgeR”. Initially, samples with zero expression were excluded. Differential expression analysis was performed to explore differences between the two groups. Finally, gene set enrichment analysis (GSEA) was performed using the KEGG and Reactome databases to clarify the signaling pathways in the locations of the differential genes.

### Immune cell infiltration analysis

Belonging to the immune structure family, TLSs are hypothesized to correlate with immune cell infiltration. Consequently, we computed the immune infiltration score for each patient’s cancer tissue employing CIBERSORT. Spearman correlation analysis was utilized to examine the association between signature genes and the immune infiltration score. Lastly, the impact of copy number variation (CNV) on immune cell infiltration was investigated through gene set cancer analysis (GSCA).

### Biological functional analysis

The “clusterProfiler” R package facilitates the functional annotation of numerous coding and non-coding genomic datasets using current gene annotations. Offering a unified interface for gene function annotations sourced from various databases, it is adaptable to diverse analytical contexts. We employed this tool to conduct gene ontology (GO) analyses for biological processes, cellular components, and molecular functions, as well as disease ontology (DO) analysis.

### Immune microenvironment analysis

Immune cell infiltration was identified using timer 2.0 (cistrome.shinyapps.io/timer/) via the MCPCOUNTER, CIBERSORT, QUANTISEQ, Timer, CIBERSORT-ABS, EPIC, and XCELL algorithms. Infiltration levels of stromal and immune cells can be calculated with the ESTIMATE algorithm^15^. Concentration scores of 16 immune cells were calculated using “GSEABase” and “GSVA” packages. The TIMER database studied six immune cells (B cells, macrophages, neutrophils, dendritic cells, CD8+ T cells, and CD4+ T cells) infiltration for its association with TLSs expression. The expression of multiple immune checkpoint molecules was compared to determine whether there were differences in immune checkpoint blockade (ICB) therapy between the TLSs+ and TLSs- groups. Immune checkpoints with differential expression between the two groups were visualized. Additionally, TIDE (Tumor Immune Dysfunction and Exclusion) score was calculated online following the instructions (https://tide.dfci.harvard.edu/). An inverse correlation was found between the TIDE score and ICB treatment success^16^.

### Antigen presentation analysis

Human leukocyte antigen (HLA), found on numerous immune cell surfaces, is crucial for triggering cellular and humoral immunity^17^. To determine whether or not there were distinctions in antigen expression between the two groups, the “limma” package was used to compare the HLA expression levels of the two groups.

### Cancer stem cell infiltration analysis

We utilized the UCSC Xena browser (http://xena.ucsc.edu/) to extract DNA methylation-based stemness scores (DNAss) and RNA-based stemness scores (RNAss) for TCGA-LUAD patients. A comparative analysis was then performed at both the DNA and RNA levels to investigate differences in stem cell infiltration between the two groups.

### Predicting drug therapeutic response

The Cancer Immunome Atlas (https://tcia.at/) was employed to derive the immunophenoscore (IPS) for predicting sensitivity to immunotherapy. Additionally, the IC50 values of common chemotherapeutic agents within the entire TCGA cohort were calculated using the “pRRophetic” software package to evaluate the predictive capacity of AGRs for drug treatment response. Subsequently, differences in IC50 values between the two groups were compared utilizing the Wilcoxon rank-sum test. Finally, the results were visualized through bar charts.

### Multiplex IHC staining

Frozen sections and formalin-fixed paraffin-embedded (FFPE) tissue sections were used. Opal 7-color kit (PerkinElmer) was used for multiplex IHC and is summarised in Extended Data Table 1. Four micrometers of FFPE sections were dewaxed and rehydrated. Human FFPE tonsil sections were used as positive controls for CD3, CD4, CD8, CD20, CD21, CD23, CK, Foxp3, TCF1, DC-LAMP, PD1, and PNAd, and lung cancer tissue was used as a negative control. CD3, CD20, CD21, CD23, DC-LAMP, and PNAd form panel 1 and mark the TLSs. The remaining antibodies formed panel 2 and labeled the TILs in the TME. Antigen retrieval using high pH (MXB) or low pH (Servicebio) antigen extracts in a microwave oven. Antibodies used for Multiplex IHC are summarised in Extended Data Table 2. For the 2 panels staining, a tyramide system amplification (TSA) was used. In the first round antigen was retrieved with a microwave oven at 100% fire for 150 s, and 30% fire for 12 min. Slides were cooled to room temperature (RT) and washed with TBST/0.5% Tween (3 times, 5 min). Slides were washed and blocked with blocking buffer (BLOCJING/AB DILUENT) for 12 min. The primary antibody was incubated at 37℃ for 1 h or 4℃ overnight. Slides were washed and an HRP-conjugated secondary antibody was incubated at 37℃ for 10 min. TSA dye (1:100) was applied for 10 min at 37℃ after washes. This was repeated five more times using the remaining antibodies. Nuclei were stained with DAPI (PerkinElmer) and mounted with a coverslip. Secondary antibodies (PerkinElmer, OPAL POLYMER HRP MS+RB) were used at an original dilution.

### Multiplex IHC imaging and inForm analysis

Slides were imaged using a Vectra microscope. Whole slide scans were performed using the × 10 objective lens. ROIs were selected with fixed-size stamps in Phenochart (PerkinElmer), based on the previously acquired whole slide scan images. A 1 × 1 stamp (669 × 500 µm;  × 20 objective lens) was employed for the Margin, while a 2 × 2 stamp (1338 × 1000 µm) was utilized for the Core, Edge, and Normal regions. To maximize viable regions in each specimen, selections were made with minimal overlap. Acquired images underwent analysis with inForm for tissue-component segmentation, distinguishing between tumor-cell (CK+) and stroma (CK-) regions, as well as cell phenotyping. The cell density within each ROI was computed by aggregating cell counts from all images and normalizing them by the total area (cells/mm^2). Multiplex IHC images were independently analyzed and blinded by three observers.

### Statistical analysis

R (version 4.2.1) was employed for all statistical analyses and graphical creations. Volcano plots were generated using the “ggplot2” package, while violin plots were created using the “ggpubr” package. The Mann–Whitney test was utilized for differential gene expression analysis, tumor mutation burden analysis, single-sample gene set enrichment analysis (ssGSEA) score analysis, immunological checkpoint analysis, and HLA analysis. Correlation tests were employed for cancer stem cell infiltration and drug sensitivity tests. Additionally, the log-rank test and Kaplan–Meier analysis were utilized to compare overall survival (OS), progression-free survival (PFS), and disease-specific survival (DSS) between groups. Unpaired two-sided t-tests were performed using Prism 9 (Graphpad) to analyze differences between groups. *P* values < 0.05 were considered statistically significant.

### Informed consent statement

We confirm that informed consent was obtained from all participants involved in this study, including the use of tissue samples. Prior to their participation, all subjects or their legal guardians were provided with detailed information regarding the nature and purpose of the study, potential risks and benefits, confidentiality measures, and their rights as participants. They were given the opportunity to ask questions and clarify any concerns before providing their consent. Written consent forms were signed by all participants or their legal guardians before any data collection or procedures were carried out. This study was conducted following the ethical principles outlined in the Declaration of Helsinki and approved by the Renmin Hospital of Wuhan University Ethics Committee.

## Results

### TLSs is associated with better survival prognosis in NSCLC patients

**Figure 1:**
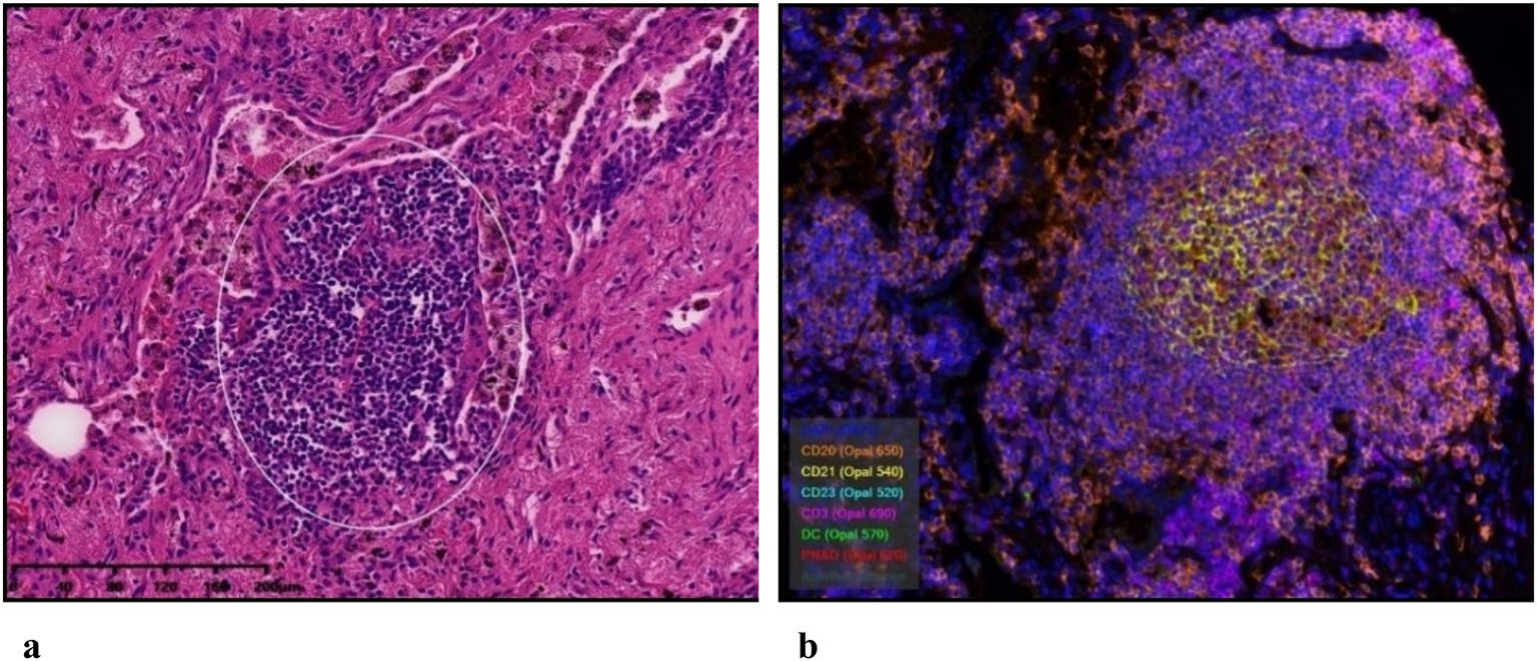
Observed TLS images. (**a**) TLS is shown in white circles in HE pathological tissue sections. (**b**) TLS representative staining by multiplex immunofluorescence: CD20 (orange), CD21 (yellow), CD23 (cyan); DC (green), CD3 (purple), PNAd (red). DAPI staining is shown in blue.

**Figure 2:**
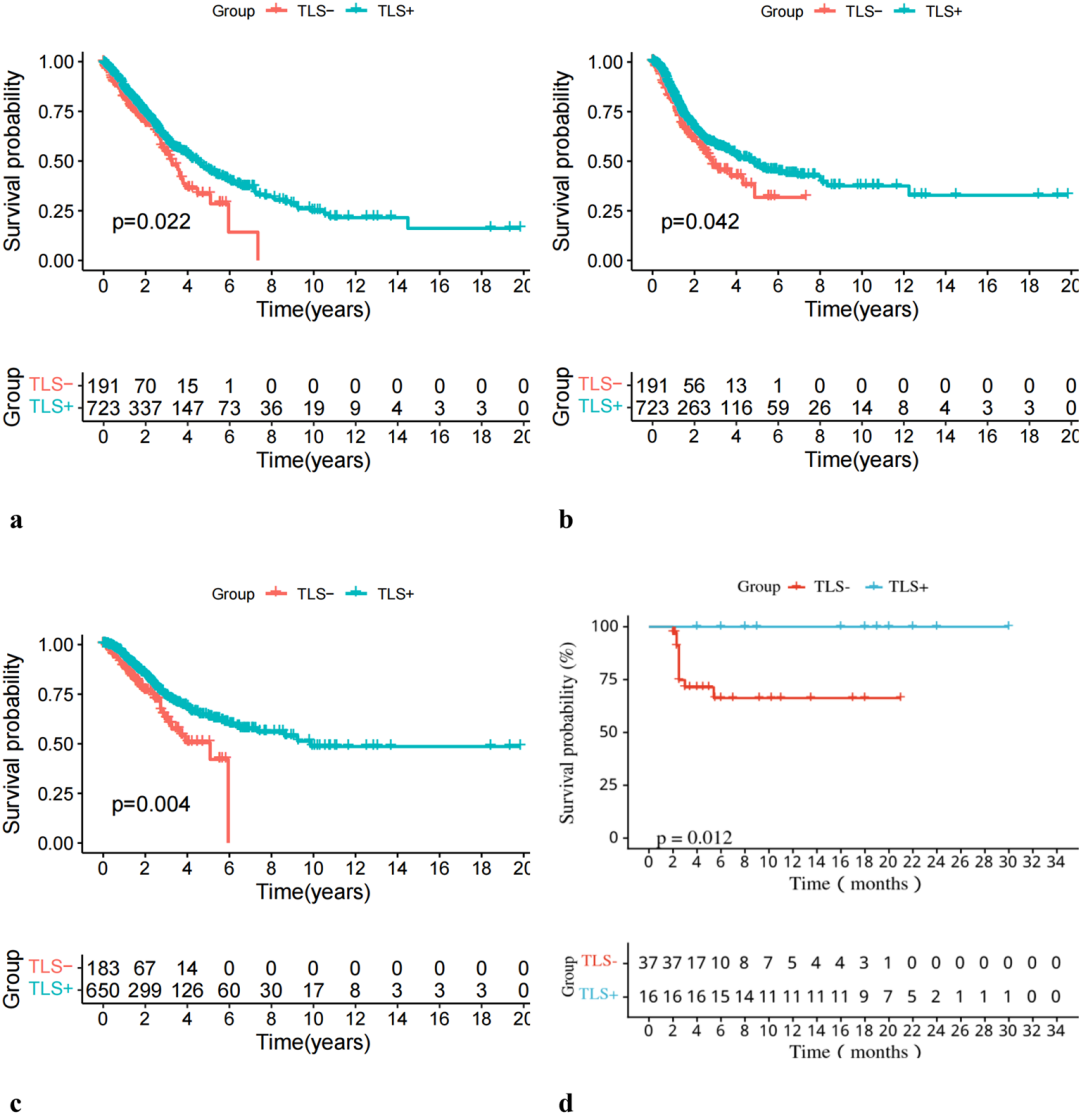
TLSs are predictive of the survival of patients with NSCLC. This figure refers to TCGA cohort (**a**–**c**) and Study cohorts (**d**). (**a**) OS curves of TLS-group vs. TLS+ group in the TCGA cohort (n = 914). (**b**) PFS curves of TLS-group vs. TLS+ group in the TCGA cohort (n = 914). (**c**) DSS curves of TLS-group vs. TLS+ group in the TCGA cohort (n = 834). (**d**) PFS curves of TLS-group vs. TLS+ group in the Study cohort (n = 53).

A total of 914 samples in the TCGA cohort were observed. There are 723 samples showing TLSs belonged to the TLS+ group, and the remaining 191 samples without lymphocytic infiltration to the TLS- group. As well, among the 53 samples in the study cohort, 16 cases belonged to the TLS+ group and 37 cases to the TLS- group (Fig. 1). Results of survival curves showed that the TLS+ group was associated with better OS (*p* = 0.022), PFS (*p* = 0.042), and DSS (*p* = 0.004) in the TCGA cohort (Fig. 2a–c). In our Study cohort, the TLS+ group also showed a better PFS (*p* = 0.012) than TLS- group (Fig. 2d). This further validates the prognostic effects of TLSs in patients with NSCLC.

### Tumor mutation burden analysis

**Figure 3:**
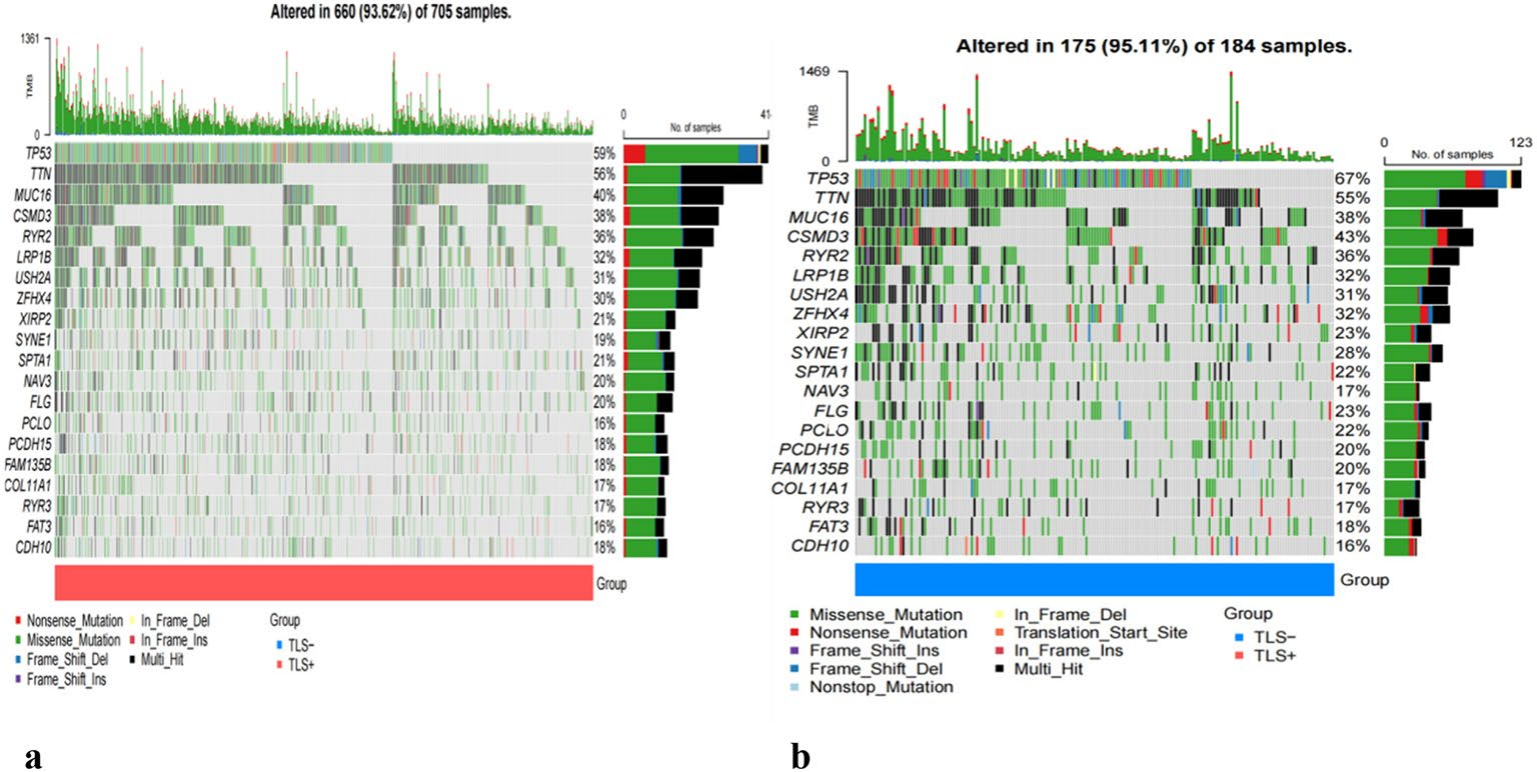
Tumor mutation burden analysis. Assessment of the differences in the mutational landscape between the TLS+ (**a**) and TLS- (**b**) groups.

We analyzed mutation rates between the TLS+ and TLS- groups. The results of the study showed a higher frequency of mutational events within the TLS- group, with mutated genes including TP53, TTN, MUC16, CSMD3, RYR2, LRP1B, USH2A, ZFHX4, XIRP2, SYNE1, SPTA1, NAV3, FLG, PCLO, PCDH15, and FAM135B. although there was no statistical difference between the two groups (TLS+ vs. TLS- 93.62% vs. 95.11%) (Fig. 3a,b).

### Immune features analysis

**Figure 4:**
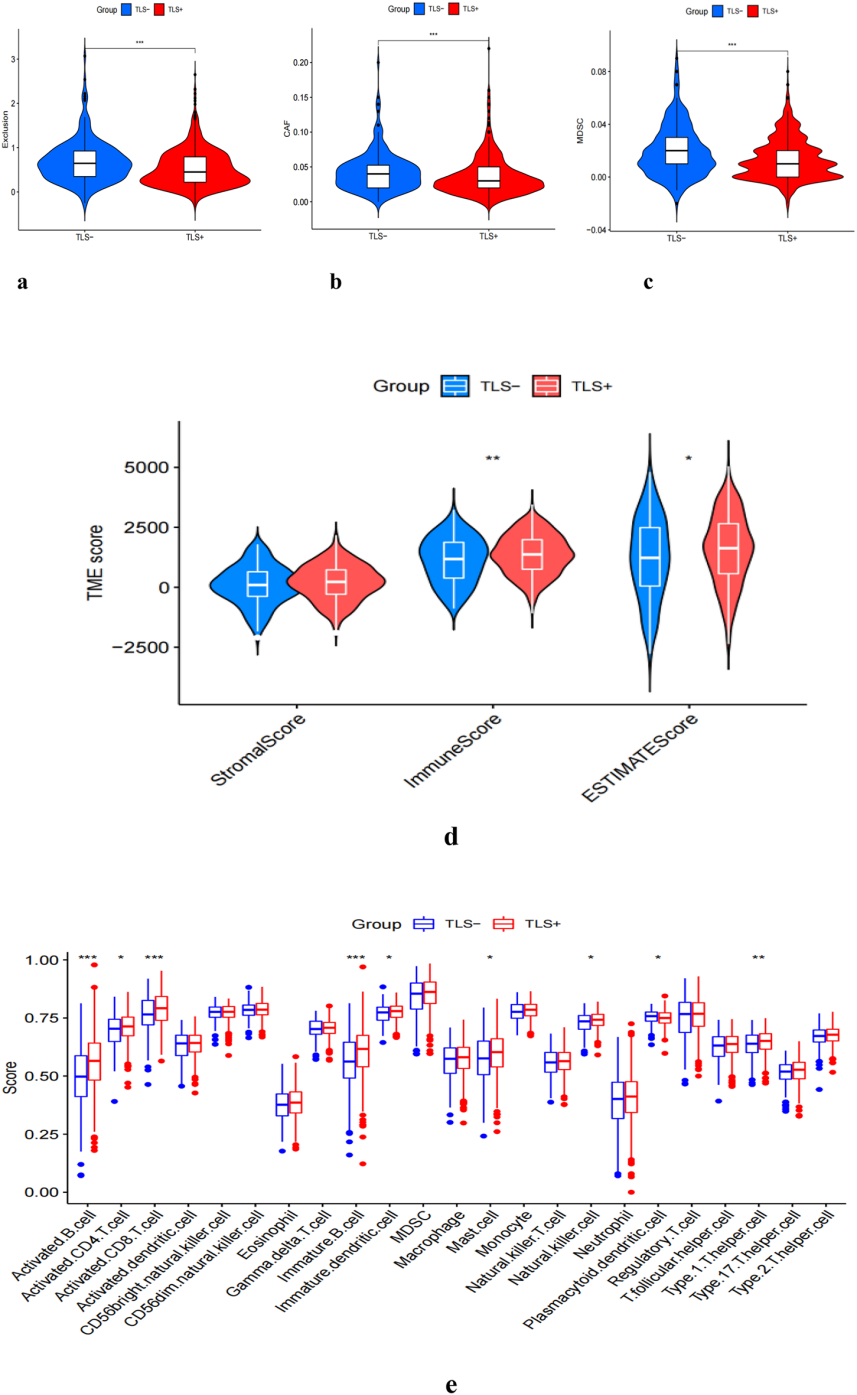

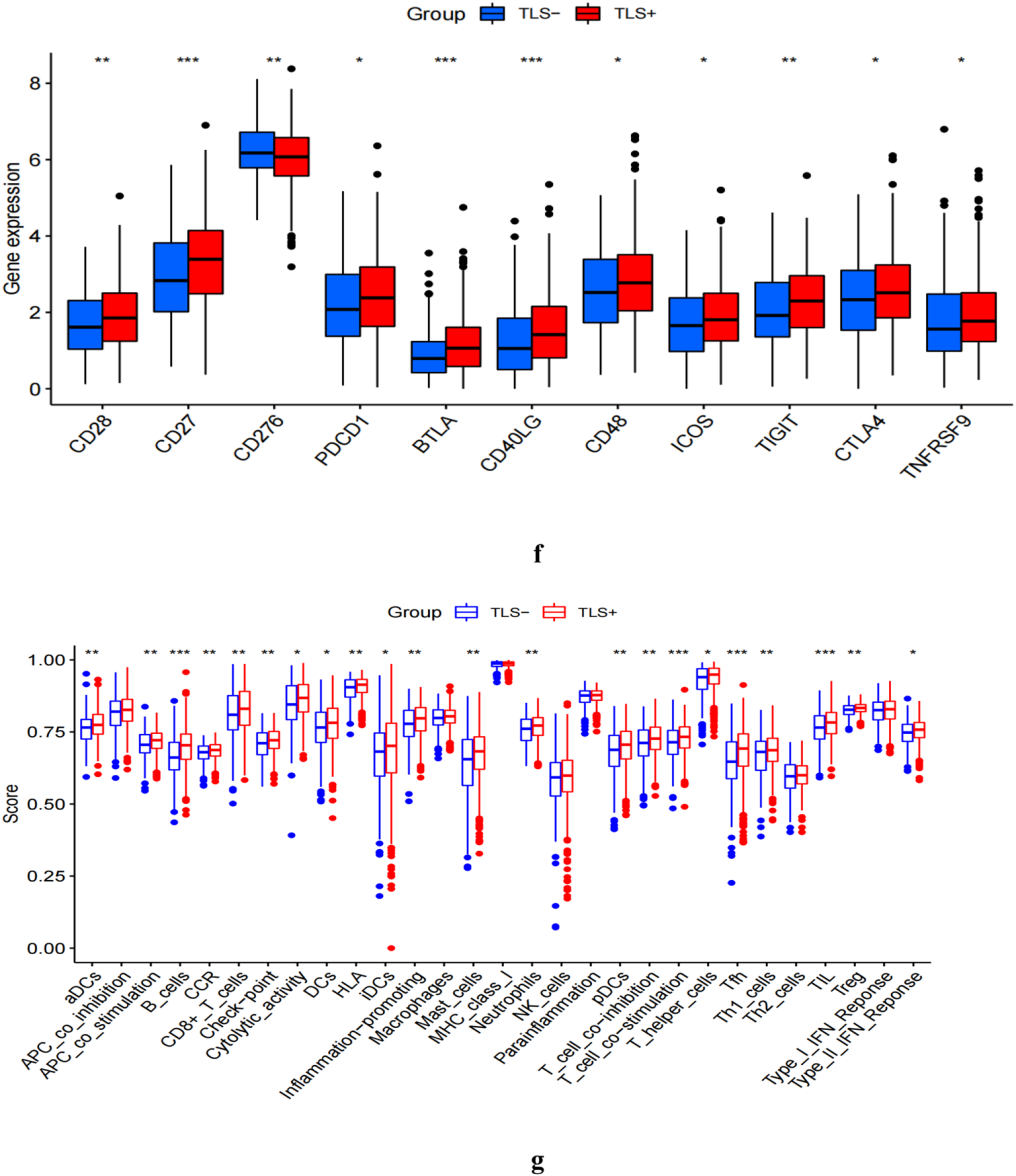
Immune features analysis. Comparison of TIDE and TLSs (**a**). Comparison of CAF and TLSs (**b**). Comparison of MDCS and TLSs (**c**). TME score in TLS+ and TLS- Groups (**d**). Immune response in TLS+ and TLS- Groups (**e**). Expression of immune checkpoints (**f**). Infiltration score of immune cells (**g**). Significance markers, ns: *p* > 0.05;*P < 0.05, **P < 0.01, ***P < 0.001.

We further explored the relationship between TLSs and the immune status of NSCLC patients in the TCGA cohort. We found that the TLS+ group had a significantly better immune status and higher levels of immune cells than the TLS- group, especially activated B cells, immature B cells, activated CD8+ T cells, and Th1 (Fig. 4e). Next, we compared the immune cell scores of the two groups in the TCGA cohort. The results showed that the infiltration scores of most immune cells in the TLS+ group were higher than in TLS- groups, such as aDCs, B cells, check-point, CD8+ T cells, and T-helper cells (Fig. 4g). Considering the importance of checkpoint inhibitors in clinical treatment, we further analyzed the differences in ICIs expression and found substantial differences in CD27, CD28, PDCD1, TIGIT, CTLA4 and BTLA between the two groups (Fig. 4f). Next, we evaluated the potential therapeutic effectiveness of immunotherapy in both patient groups using TIDE. A higher TIDE prediction score, the higher the likelihood of immune evasion, suggesting that patients are less likely to benefit from ICI therapy. We found that patients in the TLS+ group had a lower TIDE score than those in the TLS- group, suggesting that the TLS+ patients might respond better to ICI therapy in NSCLC (Fig. 4a). In addition, we also discovered that cancer-associated fibroblast (CAF) and Myeloid-derived suppressor cells (MDSC) were more excellent in the TLS- group (Fig. 4b,c). Meanwhile, patients in the TLS+ group had a significantly higher immune score in the TME (Fig. 4d).

### Gene set enrichment analyses

**Figure 5:**
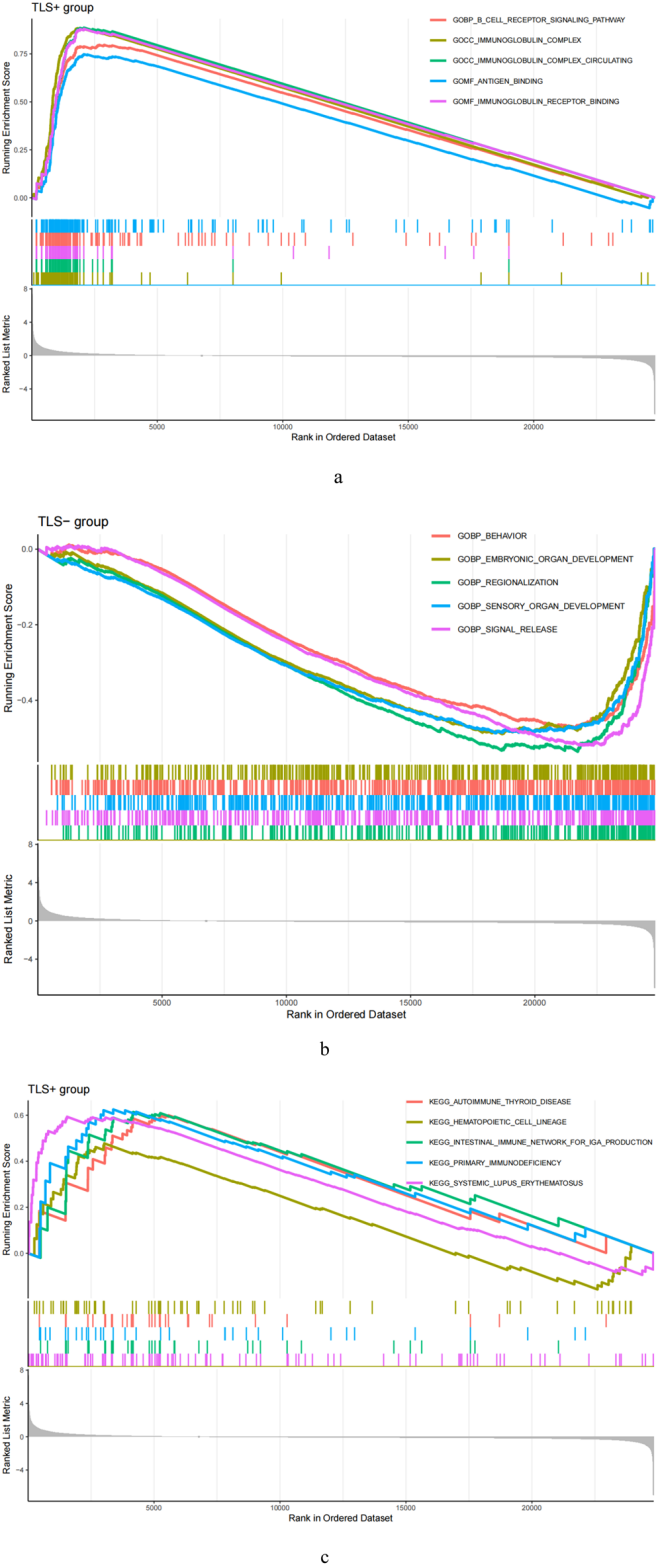

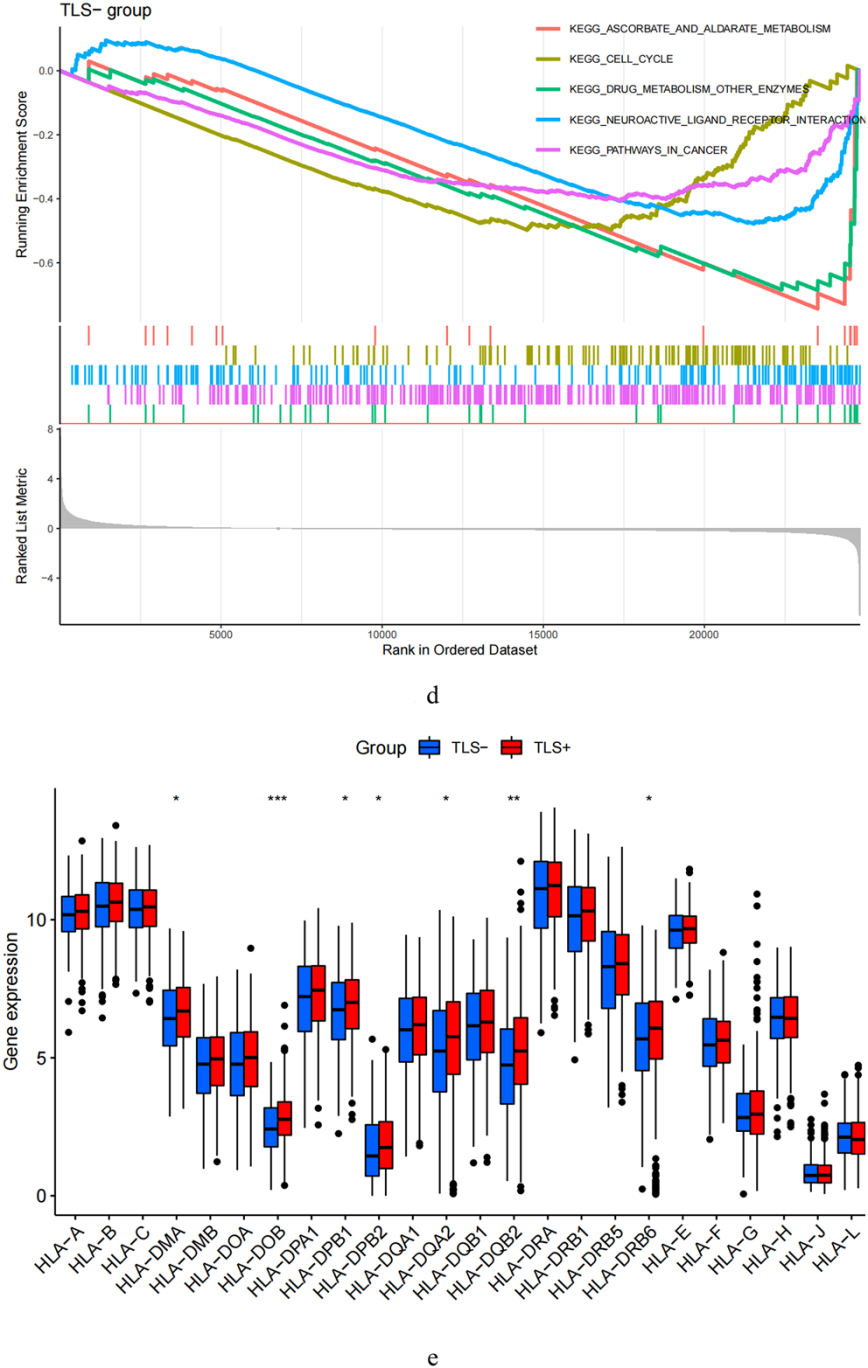
Exploration of possible reasons for differences between TLS+ and TLS- groups. (**a**, **b**) KEGG Enrichment Analysis. (**c**, **d**) GO Enrichment Analysis. (**e**) Expression of HLA. *p < 0.05, **p < 0.01, ***p < 0.001.

According to KEGG, pathways enriched in the TLS+ group included the Autoimmune thyroid disease, Haematopoietic cell lines, The intestinal immune network promotes IgA production, primary immunodeficiency, and systemic lupus erythematosus, whereas pathways enriched in the TLS- group included ascorbate and aldehyde metabolism, cell cycle, drug metabolism other enzymes, neuroactive ligand-receptor interactions and pathways in cancer (Fig. 5a,b). Additionally, GO revealed that genes in the TLS+ group were engaged in B-cell receptor signaling pathways, Immunoglobulin complex, Immune globulin complexes in circulation, Antigen binding, and Immunoglobulin receptor binding, whereas genes in the TLS-group were enriched in behavior, embryonic organ development, regionalization, sensory organ development, signal release (Fig. 5c,d).

### Antigen presentation analysis

There was a substantial variation in HLA expression associated with antigen presentation between the TLS+ and TLS- groups. The expression of numerous HLA classes I and II was more significant in the TLS+ group than in the TLS- group in the total TCGA cohort (Fig. 5e).

### Drug sensitivity analysis

**Figure 6:**
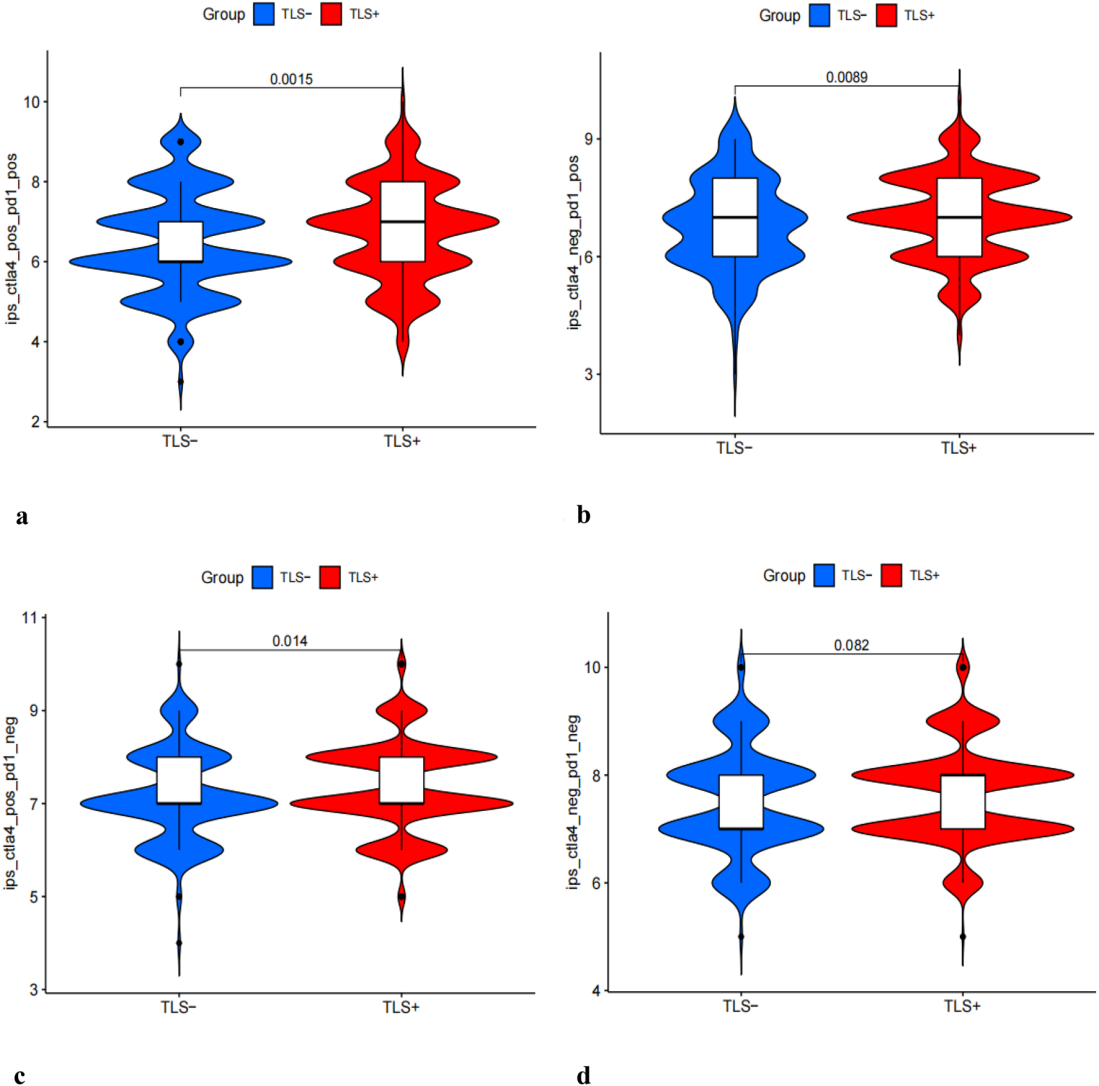
Immunotherapeutic responses of the ARGs prognostic signature and drug sensitivity analysis. Comparison of the immunophenoscore (IPS) between the TLS+ and TLS- groups stratified by both CTLA4 and PD-1 (**a**–**d**).

We followed the impact of TLS on the efficacy of immunotherapy by TCIA. The results demonstrated that the TLS+ group was more likely to respond to CTLA4-positive/PD1 markerpositive treatment than the TLS- group (Fig. 6a–d). This shows that patients in the TLS+ group may respond better to CTLA4-positive/PD1-positive immunotherapy, leading to a better clinical outcome.

### Effect of immunotherapy is associated with the size and count of TLSs in NSCLC patients

**Figure 7:**
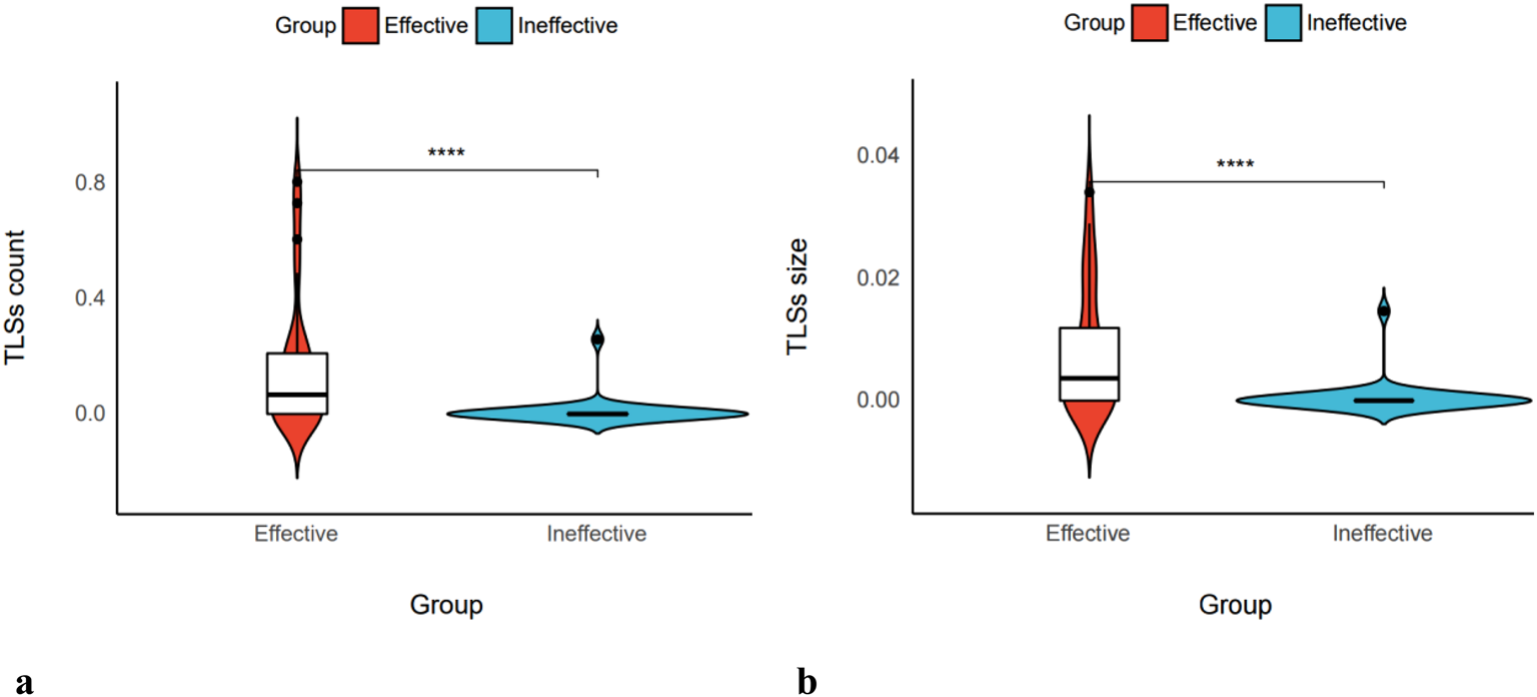
The number and size of TLSs correlate with response to immunotherapy in NSCLC patients. This result was performed in the Study cohorts. Comparison of TLS count and immunotherapy efficacy (**a**). Comparison of TLSs size and immunotherapy efficacy (**b**). two-tailed unpaired t-test for two group comparisons. Significance markers, ns: *p* > 0.05;*P < 0.05, **P < 0.01, ***P < 0.001.

To further explore the correlation between TLSs and the efficacy of immunotherapy in NSCLC, we divided the 53 cases in the study cohort into an effective and an ineffective group. Patients with a response evaluation of complete response (PR), partial response (CR), and stable disease (SD) for at least 6 months were in the effective group (n = 25). Those cases that didn’t meet this condition in the sample were in the ineffective group (n = 28). The TLSs in the above cases were quantified by counting and measuring the area of the TLSs under whole slices. The size of the TLSs was expressed by the relative area (total measured area of TLSs/area of sectioned tissue) to reduce the size error of different sections of tissue. The results showed statistically significant differences in TLS count and TLS size between samples in the Effective and Ineffective groups (*p* < 0.0001 and *p* < 0.0001, respectively; Fig. 7a,b). This indicated that the number and size of TLSs within the whole TME may contribute to the response of immunotherapy in NSCLC.

### Relationship between different T cell phenotypes and TLSs in TME

#### CD4+ T cell phenotypes

**Figure 8:**
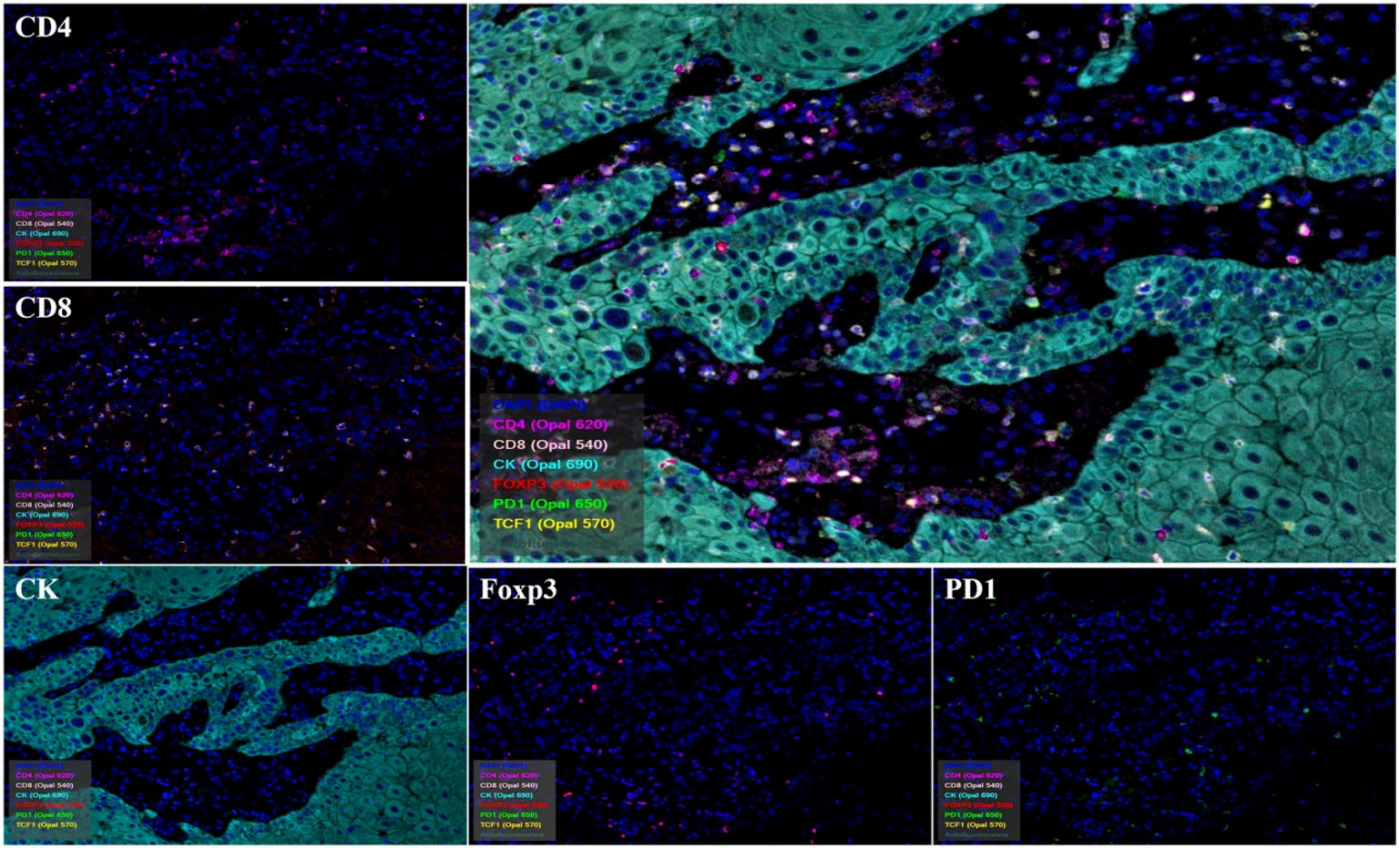
Multiplex staining of Tumor-infiltrating lymphocytes (TILs) in cancer samples. Cancer tissue was stained against CD4, PD1, FOXP3, CD8, PanCK and TCF1 using Opal 7-color Kit. The image was captured using Vectra3.0 automated quantitative pathology imaging system.

**Figure 9:**
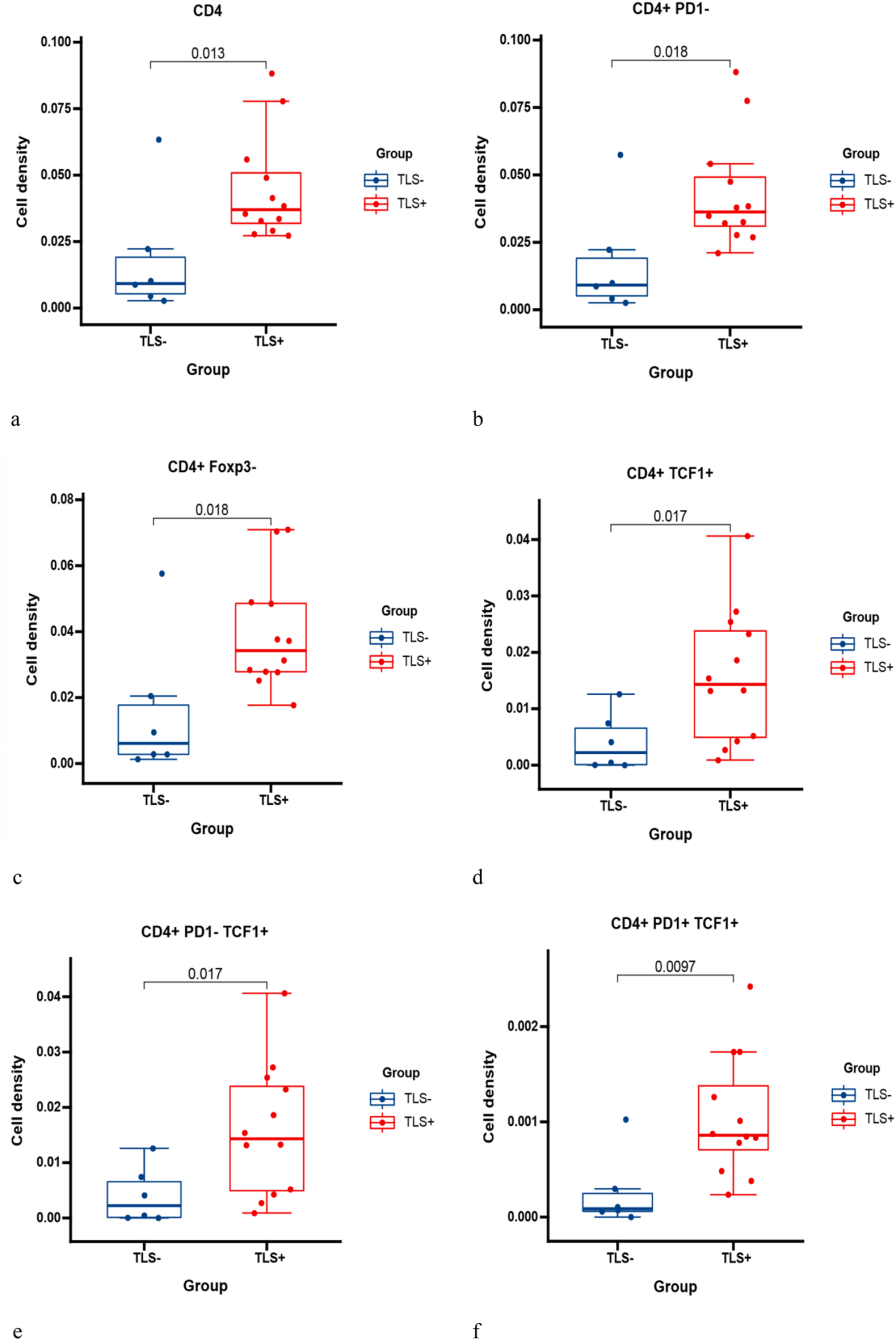
Different phenotypes of CD4+ T cells between the TLS+ and TLS- groups. Comparison of CD4+ T cells and TLSs (**a**). Comparison of CD4+PD1- T cells and TLSs (**b**). Comparison of CD4+Foxp3- T cells and TLSs (**c**). Comparison of CD4+TCF1+ T cells and TLSs (**d**). Comparison of CD4+PD1-TCF1+ T cells and TLSs (**e**). Comparison of CD4+PD1-TCF1+ T cells and TLSs (**f**).

We performed a multiplex mIHC staining on tissue sections from 18 samples in the study cohort, and simultaneous detection of CD4/CD8+ lymphocytes, CK+ tumor cells, PD1+ cells, Foxp3+ cells, and TCF1+ cells in the TME by Inform 2.6. Cell phenotyping data were obtained based on the pattern of marker expression in panel 2 (Fig. 8). Our results showed that CD4+ T cell levels were significantly higher in the TLS+ group than in the TLS- group (Fig. 9a, *p*  = 0.013). It also showed a significantly higher level of the marker TCF1 in the TLS+ group than in the TLS- group. However, the expression of markers PD1 and Foxp3 did not show differences in the two groups (Extended Fig. 2a–c). We then co-localized other markers with CD4+ T cells and found that levels including CD4+PD1-, CD4+Foxp3-, and CD4+TCF1+ T cells were significantly higher in the TLSs+ group than in the TLS- group (Fig. 9b–d), which was consistent with the results in CD4+Foxp3+ T cells, while CD4+PD1+ T cells were not significantly different between the two groups (Extended Fig. 2d,e). Besides, higher levels of CD4+PD1-TCF1+ T cells and CD4+PD1+TCF1+ T cells were observed in the TLS+ group (Fig. 9e,f). This suggests that TLSs are more closely associated with CD4+ helper T cells in TME.

#### CD8+ T cell phenotypes

**Figure 10:**
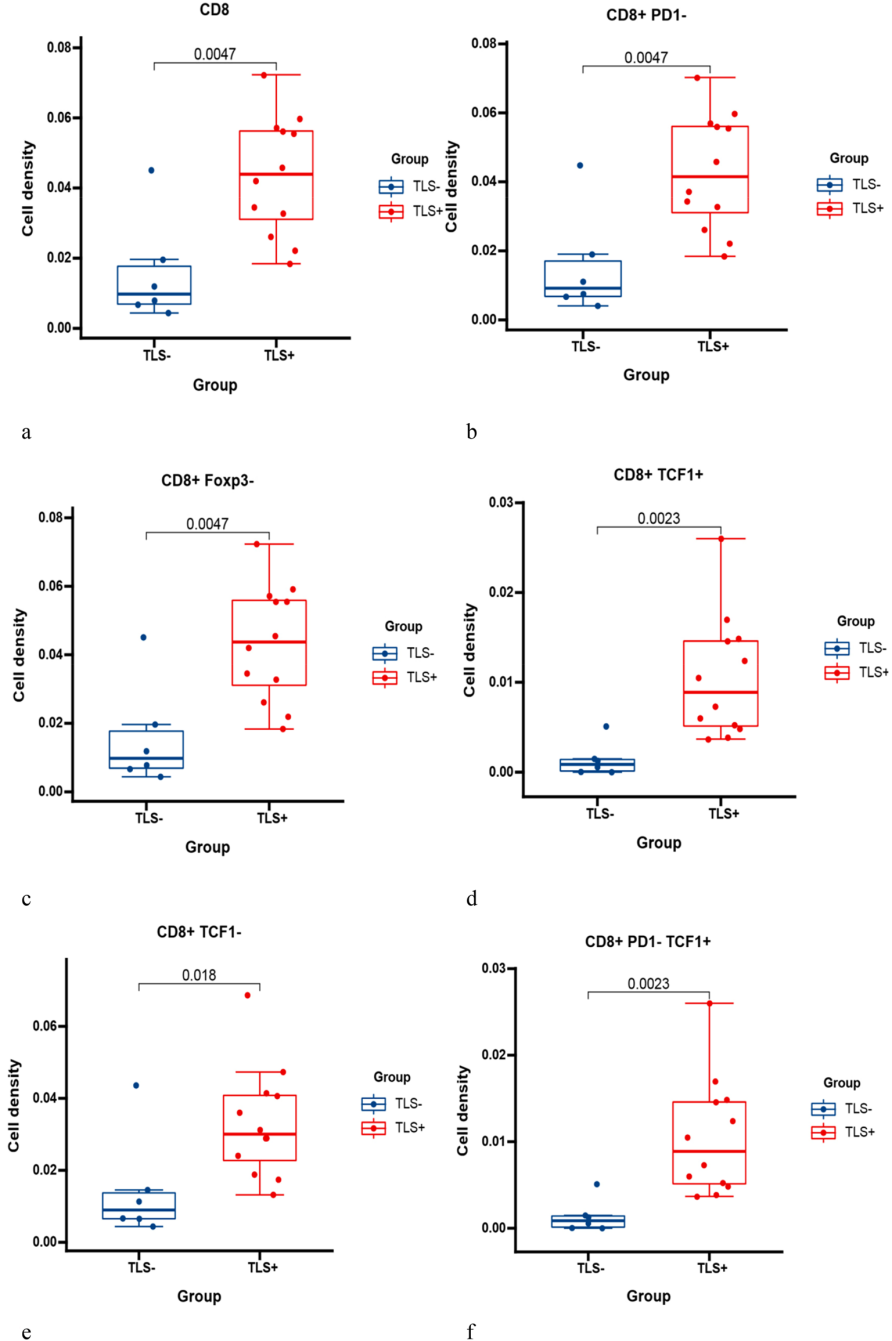
Different phenotypes of CD8+ T cells between the TLS+ and TLS- groups. Comparison of CD8+ T cells and TLSs (**a**). Comparison of CD8+PD1- T cells and TLSs (**b**). Comparison of CD8+Foxp3- T cells and TLSs (**c**). Comparison of CD8+TCF1+ T cells and TLSs (**d**). Comparison of CD8+TCF1- T cells and TLSs (**e**). Comparison of CD8+PD1-TCF1+ T cells and TLSs (**f**).

Naturally, we analyzed the CD8+ T cell with different markers in the same way. The results showed that CD8+ T cell levels were significantly higher in the TLS+ group than in the TLS- group (Fig. 10a, *p*  = 0.0047). Then, we found that the level of CD8+ T cells was significantly higher in the TLSs+ group than in the TLS- group, such as CD8+PD1- (*p* = 0.0047), CD8+Foxp3- (*p* = 0.0047) and CD8+TCF1+ (*p* = 0.0023) T cells (Fig. 10b–d). Meanwhile, CD8+Foxp3+ T cells were not clearly differentiate between the two groups (Extended Fig. 2g). Our results also indicated that CD8+PD1-TCF1+ T cells with stem cell properties (mentioned in our previous study^18^) were significantly higher in the TLS+ group than in the TLS- group (Extended Fig. 2h), while CD8+PD1+ T cells were not significantly different between the two groups (Extended Fig. 2f).

## Discussion and conclusion

The advent of ICIs has opened up new horizons in cancer therapy, and since the first antibody to block the immune checkpoint CTLA-4 was approved, anti-PD-1/PD-L1 antibodies are now the most common anti-tumor immunotherapy. However, patients rarely benefit for long in the clinic^19^. Thus, reliable predictors of immunotherapy are urgently needed.

TLSs are an important component of the immune response occurring in the tumor microenvironment and are associated with better prognosis and immunotherapy efficacy^20,21^. However, it is difficult to predict the prognosis and the efficacy of immunotherapy from TLSs alone. As such, there is an essential need to find accurate biomarkers for TLSs, which are formed by the aggregation of immune components such as B-lymphocyte clusters, T-lymphocyte clusters, macrophages, and dendritic cells (DCs), providing an orderly and efficient site for T cell proliferation and differentiation^5,6^. In studies of NSCLC^22,23^, colorectal^24,25^, gastric^26^, and ovarian cancers^27^, researchers have found that high levels of B cells or DCs within TLSs are associated with better OS through analysis of the cellular composition of TLSs. In addition, other components within TLSs have been found to have positive prognostic value, including HEVs^28^, Chemokine-12^29^ and CD3+ T cells^22^. However, it is unclear how TLSs regulate the immune response and how immune cells (particularly B cells and T cells) interact with each other^30^. Therefore, it is necessary to study the integrated role of TLSs in TME.

In our study, we included 53 NSCLC patients treated with PD1 inhibition as the study cohort. Then we obtained the dataset of 914 NSCLC pathological sections archived by CDSA from the TCGA database as the TCGA cohort and collected the corresponding transcript expression data and clinical data. When selecting specimens from the TCGA database for analysis in this study, our goal was to observe the trend of TLS-associated mutations across the entire spectrum of NSCLC cancers, including early-, intermediate-, and late-stage patients, so that 723 of 914 samples belonged to the TLS+ group; In contrast, in our own study cohort specimen, only patients with advanced NSCLC were included, whose immune status and prognosis would be somewhat worse, so only 16 of the 53 samples belonged to the TLS+ group. Our results suggest that the presence of TLSs is associated with a better prognosis, which is consistent with the previously mentioned results. Furthermore, the enrichment of TLSs was positively correlated with the number of mutations/neoantigen load, with tumors with high mutation or neoantigen load, such as melanoma, squamous lung cancer, and lung adenocarcinoma, exhibiting high expression of TLSs^14^. Paradoxically, further analysis of the mutational information obtained from the DNA sequencing data through the TCGA database revealed that the TLSs-group had a higher mutation frequency compared to the TLS+ group. We then further demonstrated that the TLS+ group is more closely related to the immune system in terms of cellular infiltration, biological function, and signaling pathways. The immune infiltration score showed that TLSs are closely associated with the infiltration of immune cells, such as CD8+ T cells, Tregs, CD4+ T cells, and NK. These cells play different roles in the anti-tumor immune response. CD4+ helper T cells promote the immune response. In contrast, Foxp3, a signature molecule of regulatory T cells (Treg), determines function of Treg and leads to Immune suppression^31,32^. We analyzed the expression of immune checkpoints in the TCGA cohort and found that most immune checkpoints were higher expressed in the TLS+ group. This further validates the predictive role of TLSs on the efficacy of immunotherapy. More importantly, we found that the count and size of TLSs in our study cohort correlated closely with the efficacy of immunotherapy in NSCLC. As a study also found in lung cancer, high-scoring TLSs showed an improved response to immunotherapy^33^.

We analyzed the immune cell components of the TME in the study cohort by mIHC and found that the expression of CD4+Foxp3+ Treg and CD4+Foxp3- helper T cells were higher in the TLS+ group than in the TLS- group. The interaction of these T cells maintains the stability of the TME, leading to a more orderly anti-tumor immune response. Therefore, the functional status of immune cells in the TME is very important. In response to prolonged high antigen load, some CD8+ effector T cells have a reduced capacity to secrete cytokines while expressing a large number of inhibitory receptors, such as PD1, T cell immunoglobulin and mucin-domain containing-3 (Tim-3), Lymphocyte activation gene 3 (LAG-3) and CTLA4. This condition is known as “T-cell failure”^34,35^. Our results show that TLSs are associated with high levels of CD8+PD1- T cells and CD4+PD1- T cells. In addition, CD8+TCF1+ T cells were also significantly highly expressed in the TLS+ group. TCF1, a T cell-specific transcription factor, is essential for early T cell development and is a downstream effector molecule of the classical Wnt signaling Pathway^36^. TCF1 was found to be essential for the self-replication of CD8+ memory T cells in the tumour microenvironment and to promote response to immune checkpoint inhibitors in tumour patients^37,38^. The low expression of inhibitory receptors in the immune microenvironment and the high expression of TCF1 may represent a T cell state that is the opposite of “T cell failure”^39^. Furthermore, our results showed that CD8+PD1-TCF1+ T cells were significantly higher expressed in the TLS+ group, and it is more beneficial to the survival prognosis and immunotherapeutic efficacy of NSCLC patients in our previous study. This also suggested that TME with TLSs indicates a more active immune state. Consistently, a previous study of sarcoma also suggests that the high immune group (E group) showed improved survival and response to PD1 blockade therapy^8^.

Naturally, there are some limitations to our study. Primarily, the pathologic specimens in both the TCGA database and our own study cohort were large specimens of single pathology sections, which provide a more comprehensive picture of the patient’s immune microenvironment and TLS than limited microarray specimens. However, due to the difficulty of obtaining specimens as well as the difficulty and cost of the test manipulation, there have been fewer studies that have chosen large samples for exploration. In order to obtain more accurate data, we chose a pathologic large sample. The source of our specimens was patients with advanced NSCLC who received immunotherapy. Previous studies on TLS did not mention the treatment of the patients and few immunotherapy patients were seen, only 2 cohort studies on sarcoma and renal cancer were published simultaneously in Nature 2020, involving immunotherapy, and similar studies on NSCLC were not seen; most importantly, we further explored the size of TLS in the TME of these advanced NSCLC patients by mIHC and number in relation to immunotherapy efficacy, and also explored the expression of TLS and T-cell surface molecules. This also further increased the difficulty of specimen collection. Furthermore, when we initially selected specimens from the TCGA database for the original letter analysis, we wished to study the trend of TLS-associated mutations across the entire spectrum of NSCLC cancers, and therefore did not differentiate between patients’ tumor stages. Instead, the specimens in our study cohort were intended to further explore the efficacy of immunotherapy, and therefore the specimens that could be obtained were from patients with advanced NSCLC. It is also due to the scarcity of pathological specimens and the difficulty of obtaining tissues from this group of patients that led to the small number of qualified samples we were able to collect, which is a shortcoming of this experiment, and we will continue to collect and expand the sample size in the clinic at a later stage.

In summary, we found that TLSs were a strong predictor of survival and immunotherapy efficacy in NSCLC patients, both the count and size of TLS correlate with the efficacy of immunotherapy. In addition, TLSs were closely associated with both CD4+ T cells and CD8+ T cells in the tumor microenvironment. T cells closely associated with TLS+, such as CD4+Foxp3+ regulatory T cells, and CD4+Foxp3- helper T cells maintain homeostasis of the immune microenvironment and contribute to the anti-tumor immune response of CD8+ effector T cells. Moreover, TLS+ is also associated with higher levels of PD1-TCF1+ stem cell-like effector T cells, which act in concert to activate a sufficiently powerful anti-cancer killing force.

### Supplementary Information

Supplementary Information.

## Data Availability

The datasets referred to in this study can be found in the online repository. Detailed information is included in the article/supplementary material. Further enquiries can be directed to the corresponding author.
