# Supplementary material for: The impact of tertiary lymphoid structures on tumor prognosis and the immune microenvironment in non-small cell lung cancer
Source: Sci Rep. 2024 Jul 15;14:16246. doi: 10.1038/s41598-024-64980-y (PMC11250816; doi:10.1038/s41598-024-64980-y)
Supplement: Supplementary file 1 — Supplementary Information. [file 41598_2024_64980_MOESM1_ESM.docx]

Extended Data Table 1

Opal-7-color Multiplex IHC KIT (Perkin Elmer)

| Description | Concentration |
| --- | --- |
| 1×PLUSAM-PLIFICATION DILUENT | / |
| OPAL520 FLUORO-PHORE | 1:100 |
| OPAL540 FLUORO-PHORE | 1:100 |
| OPAL570 FLUORO-PHORE | 1:100 |
| OPAL620 FLUORO-PHORE | 1:100 |
| OPAL650 FLUORO-PHORE | 1:100 |
| OPAL690 FLUORO-PHORE | 1:100 |
| OPAL POLYMER HRP MS+RB | / |
| BLOCJING/AB DILUENT | / |
| SPECTRAL DAPI SOLUTION | 1:10 |

Extended Data Table 2

Antibodies used for immunofluorescence

| Antibody | Source | Concentration | Secondary Antibody | Fluorescent Agent | panel |
| --- | --- | --- | --- | --- | --- |
| CD20 | MXB | 1:1 | Polymer HRP Ms+Rb | OPAL650 | 1 |
| CD21 | Dako | 1:2 | Polymer HRP Ms+Rb | OPAL540 | 1 |
| PNAd | Santa Cruz Biotechnology | 1:400 | Polymer HRP Ms+Rb | OPAL620 | 1 |
| CD23 | Dako | 1:2 | Polymer HRP Ms+Rb | OPAL520 | 1 |
| CD3 | Dako | 1:2 | Polymer HRP Ms+Rb | OPAL690 | 1 |
| DC-LAMP | Invitrogen | 1:400 | Polymer HRP Ms+Rb | OPAL570 | 1 |
| PD1 | CST | 1:1 | Polymer HRP Ms+Rb | OPAL650 | 2 |
| CD8 | Dako | 1:1 | Polymer HRP Ms+Rb | OPAL540 | 2 |
| CD4 | Dako | 1:1 | Polymer HRP Ms+Rb | OPAL620 | 2 |
| FOXP3 | CST | 1:400 | Polymer HRP Ms+Rb | OPAL520 | 2 |
| CK | Dako | 1:2 | Polymer HRP Ms+Rb | OPAL690 | 2 |
| TCF1 | CST | 1:200 | Polymer HRP Ms+Rb | OPAL570 | 2 |

Extended Data Figure 1

| **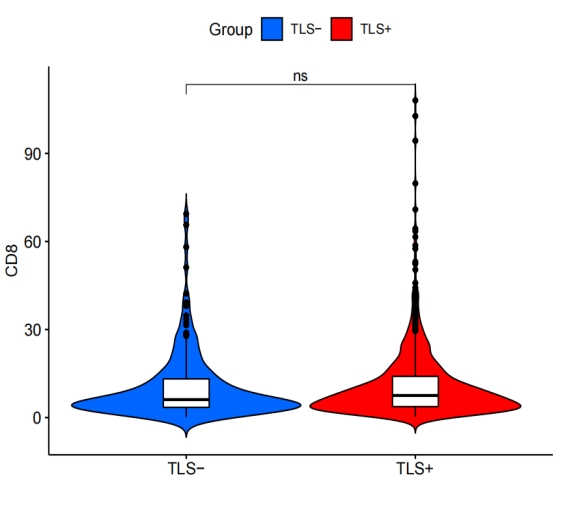**  a | **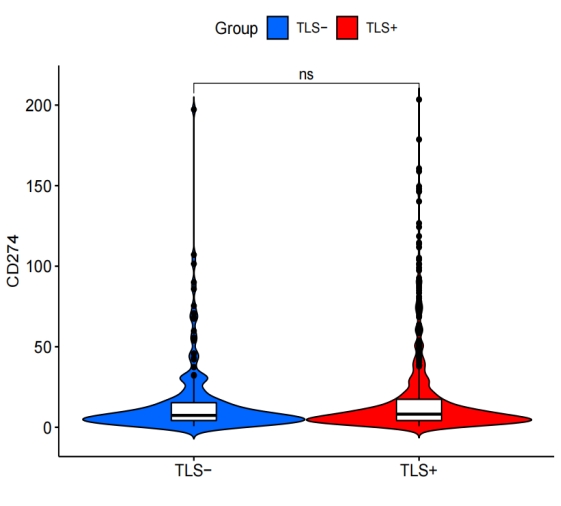**  b |
| --- | --- |
| **Figure 1.**  Immune features analysis. Comparison of CD8+ T-cell and TLSs (a). Comparison of CD274 and TLSs (b). significance markers, ns: p> 0.05;*P< 0.05, **P< 0.01, ***P< 0.001. | |
|  | |

Extended Data Figure 2

| 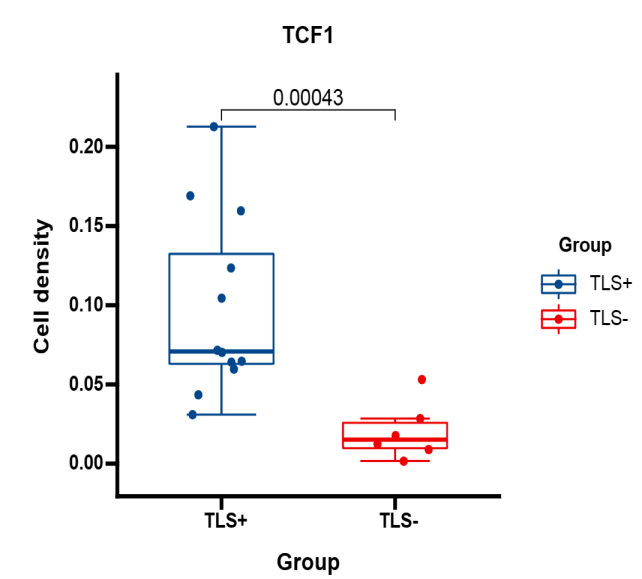  a | 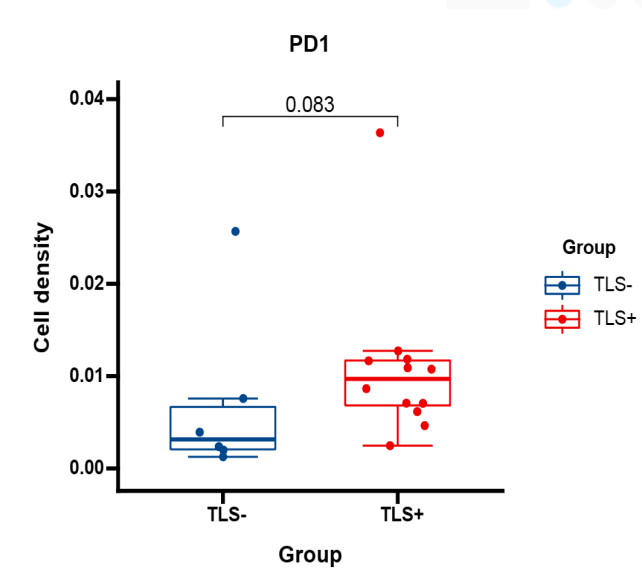  b |
| --- | --- |
| 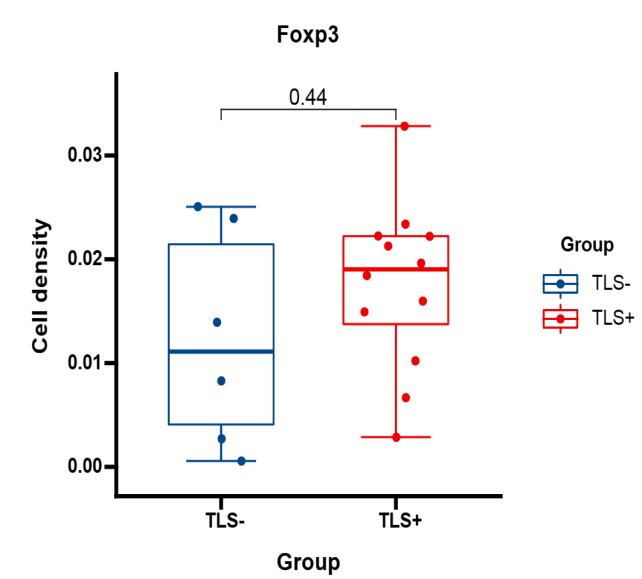  c | 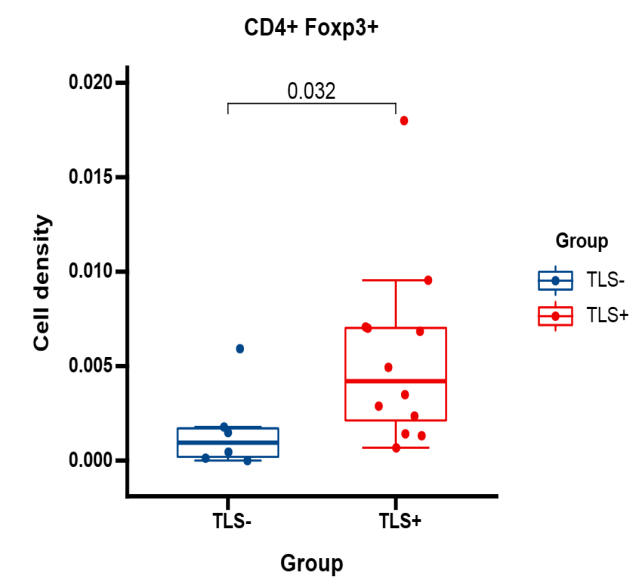  d |
| 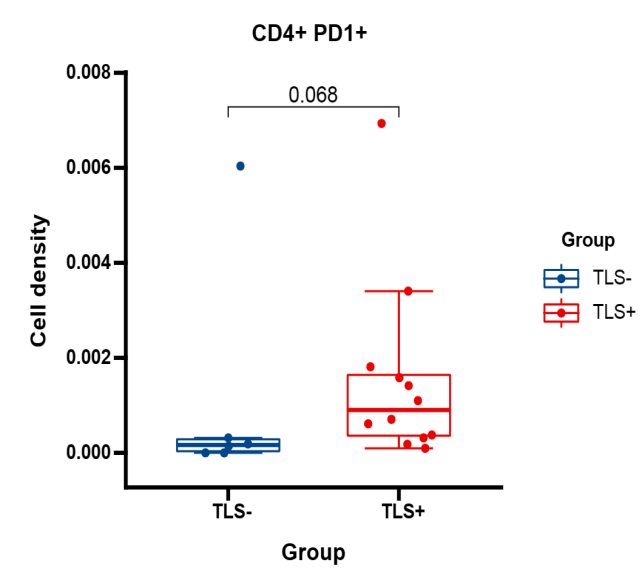  e | 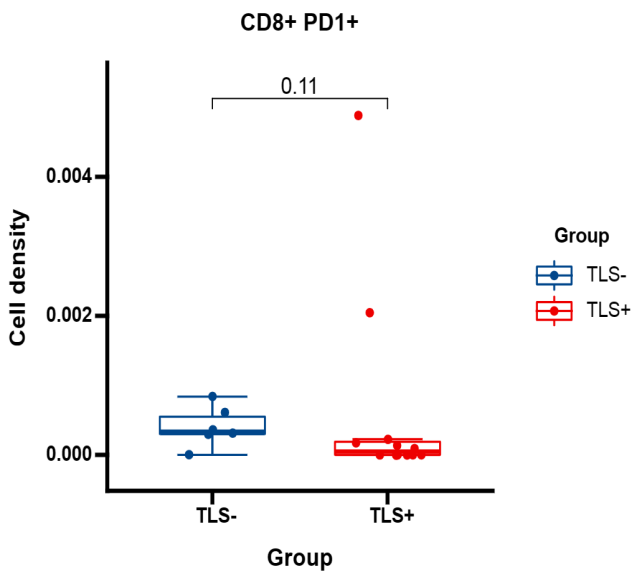  f |
| 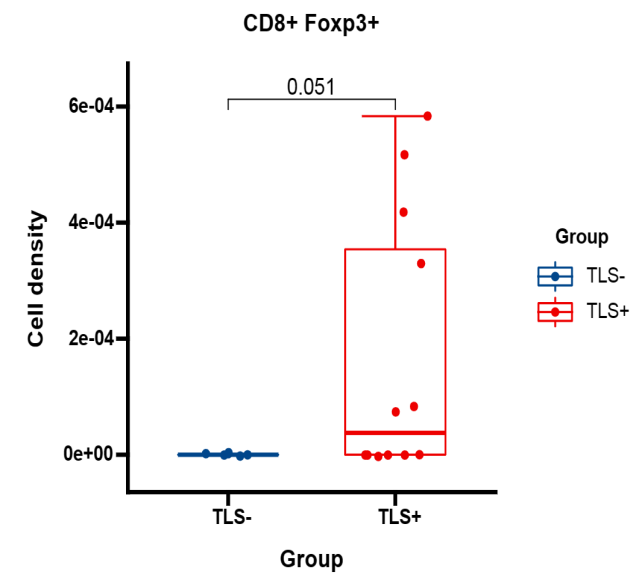  g | 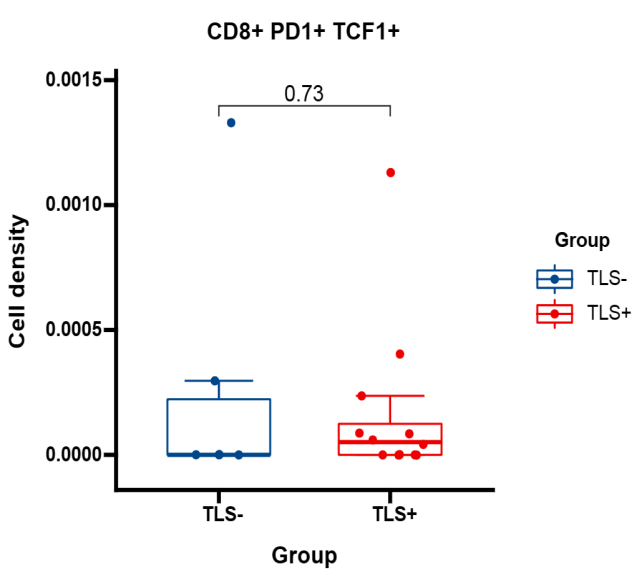  h |
| **Figure 2.**  Different markers of cells between the TLS+ and TLS- groups. Comparison of TCF1+ cells and TLSs (a). Comparison of PD1+ cells and TLSs (b). Comparison of Foxp3+ cells and TLSs (c). Comparison of CD4+Foxp3+ T cells and TLSs (d). Comparison of CD4+PD1+ T cells and TLSs (e). Comparison of CD8+PD1+ T cells and TLSs (f). Comparison of CD8+Foxp3+ T cells and TLSs (g). Comparison of CD8+PD1+ TCF1+ T cells and TLSs (h). | |
